# Severe haemorrhagic diathesis due to acquired hypofibrinogenemia in a patient with early T-cell precursor acute lymphoblastic leukaemia/lymphoma: a case report

**DOI:** 10.3389/fcvm.2023.1335296

**Published:** 2024-01-08

**Authors:** Luca Spiezia, Marcello Riva, Carmela Gurrieri, Elena Campello, Paolo Simioni

**Affiliations:** ^1^ General Internal Medicine & Thrombotic and Haemorrhagic Diseases Unit, Department of Medicine, Padova University School of Medicine, Padova, Italy; ^2^ Haematology and Clinical Immunology Unit, Department of Medicine, Padova University School of Medicine, Padova, Italy

**Keywords:** haemorrhagic diathesis, acquired hypofibrinogenemia, early T-cell precursor acute lymphoblastic leukaemia/lymphoma, case report, consumptive coagulopathy

## Abstract

The most frequent haematological malignancy associated with acquired hypo/dysfibrinogenemia is multiple myeloma. We present an unusual case of severe haemorrhagic diathesis due to acquired hypofibrinogenemia in a patient with early T-cell precursor acute lymphoblastic leukaemia/lymphoma (ETP-ALL/LBL). A 57-year-old male was admitted to the General Internal Medicine Department of Padova University Hospital for acute massive haematomas of the left lower extremity associated with macrohaematuria. Coagulation tests showed prolonged prothrombin time, activated partial thromboplastin time and thrombin time due to isolated severe hypofibrinogenemia (antigen 0.70 g/L and activity 26%). The radiological workup showed a bulky lesion located in the anterior mediastinum, and a biopsy led to the diagnosis of ETP-ALL/LBL. Fibrinogen replacement therapy failed to correct the bleeding diathesis and we were able to exclude other frequent causes of acquired hypofibrinogenemia (i.e., liver dysfunction, fibrinogen-specific antibody or drug toxicity); therefore, we hypothesised that hypofibrinogenemia might stem either from enhanced removal of fibrinogen from the circulation or consumptive coagulopathy. Notably, only after initiating a specific chemotherapy treatment did the patient start showing improvement in bleeding symptoms and achieve normal fibrinogen levels.

## Introduction

Human fibrinogen is a dimeric glycoprotein with a molecular weight of approximately 340 kDa (1), which consists of two identical halves synthesised exclusively by the liver. The conversion of soluble fibrinogen into insoluble fibrin is catalysed by thrombin, and it plays a pivotal role in clot formation and stabilisation. Moreover, fibrinogen promotes platelet aggregation in primary haemostasis by binding to platelets and contributes to tissue repair by binding to endothelial cells. Fibrinogen is also an acute phase protein whose plasma levels may increase up to fourfold during an inflammatory response (2–4).

Fibrinogen defects can be inherited due to genetic mutations or acquired as the consequence of underlying diseases (5, 6), and they manifest as reduced levels of normal fibrinogen (e.g., hypofibrinogenemia) or structural abnormalities resulting in abnormal fibrinogen function (e.g., dysfibrinogenemia). The most frequent mechanisms proposed as possible causes of acquired hypo/dysfibrinogenemia are: (*i*) consumptive coagulopathy (e.g., disseminated intravascular coagulation) (7); (*ii*) haemodilution after blood loss (e.g., trauma-induced coagulopathy) (8); (*iii*) reduced liver synthetic function; (*iv*) fibrinogen-specific antibody (9); (*v*) drug toxicity (e.g., mithramycin, L-asparaginase, tigecycline, valproic acid) (10–13). An increasing number of studies have reported an association between acquired hypo/dysfibrinogenemia and both solid and haematological tumours (14–20). Among the latter, multiple myeloma is the most frequent disease wherein the hypothesised pathophysiological mechanism underlying hypofibrinogenemia involves the presence of a paraprotein that can bind to fibrinogen and/or fibrin, thus interfering with its expression and polymerisation, and inhibiting its function (9, 21, 22).

We report the case of a patient with severe haemorrhagic diathesis due to acquired hypofibrinogenemia which led to the diagnosis of early T-cell precursor acute lymphoblastic leukaemia/lymphoma (ETP-ALL/LBL).

## Case report

**Figure 1:**
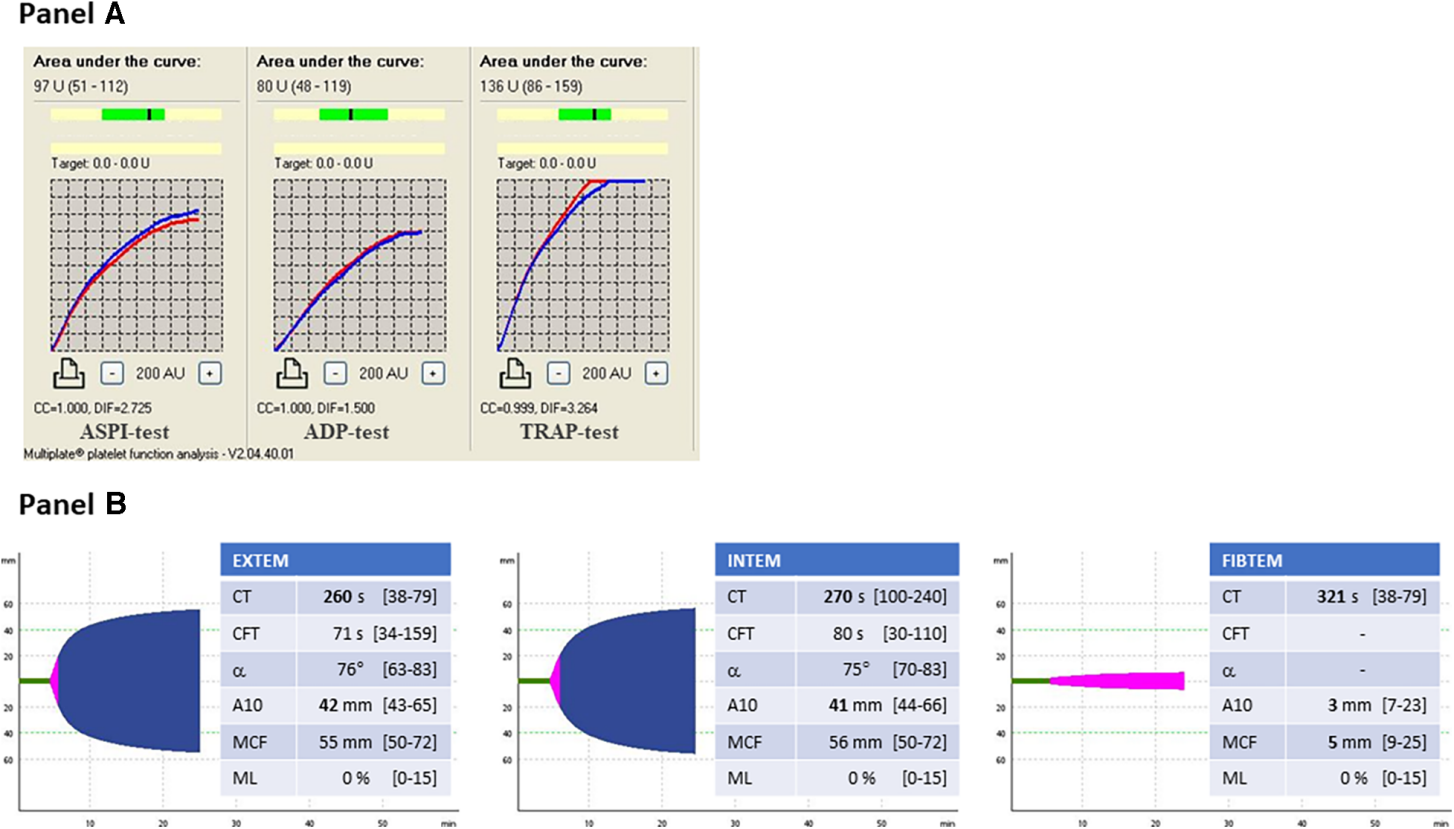
Panel (**A**) multiple electrode aggregometry profiles. ASPI-test, arachidonic acid induced aggregation; ADP-test, adenosine-diphosphate induced aggregation; TRAP-test, thrombin receptor activating peptide induced aggregation. Panel (**B**) Thromboelastometry profiles. EXTEM, evaluation of the extrinsic coagulation pathway; INTEM, evaluation of the intrinsic coagulation pathway; FIBTEM, evaluation of the contribution of fibrinogen to blood clotting. CT, clotting time; CFT, clot formation time; α, α-angle; MCF, maximum clot firmness; ML, maximum lysis.

**Figure 2:**
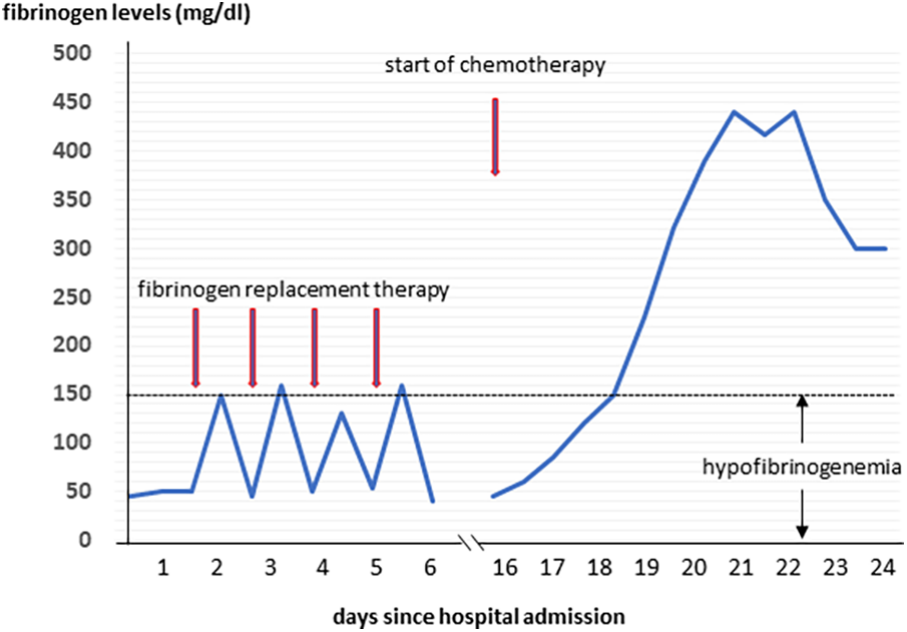
The levels of fibrinogen during hospitalization.

**Figure 3:**
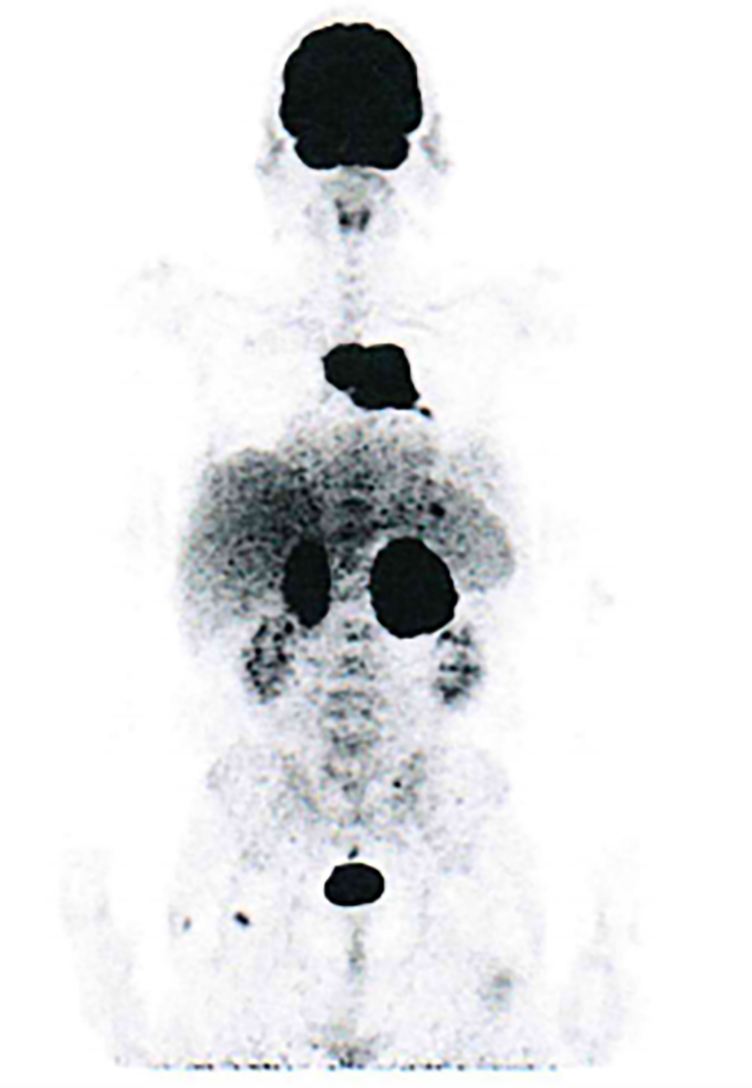
PET/MRI scan.

**Figure 4:**
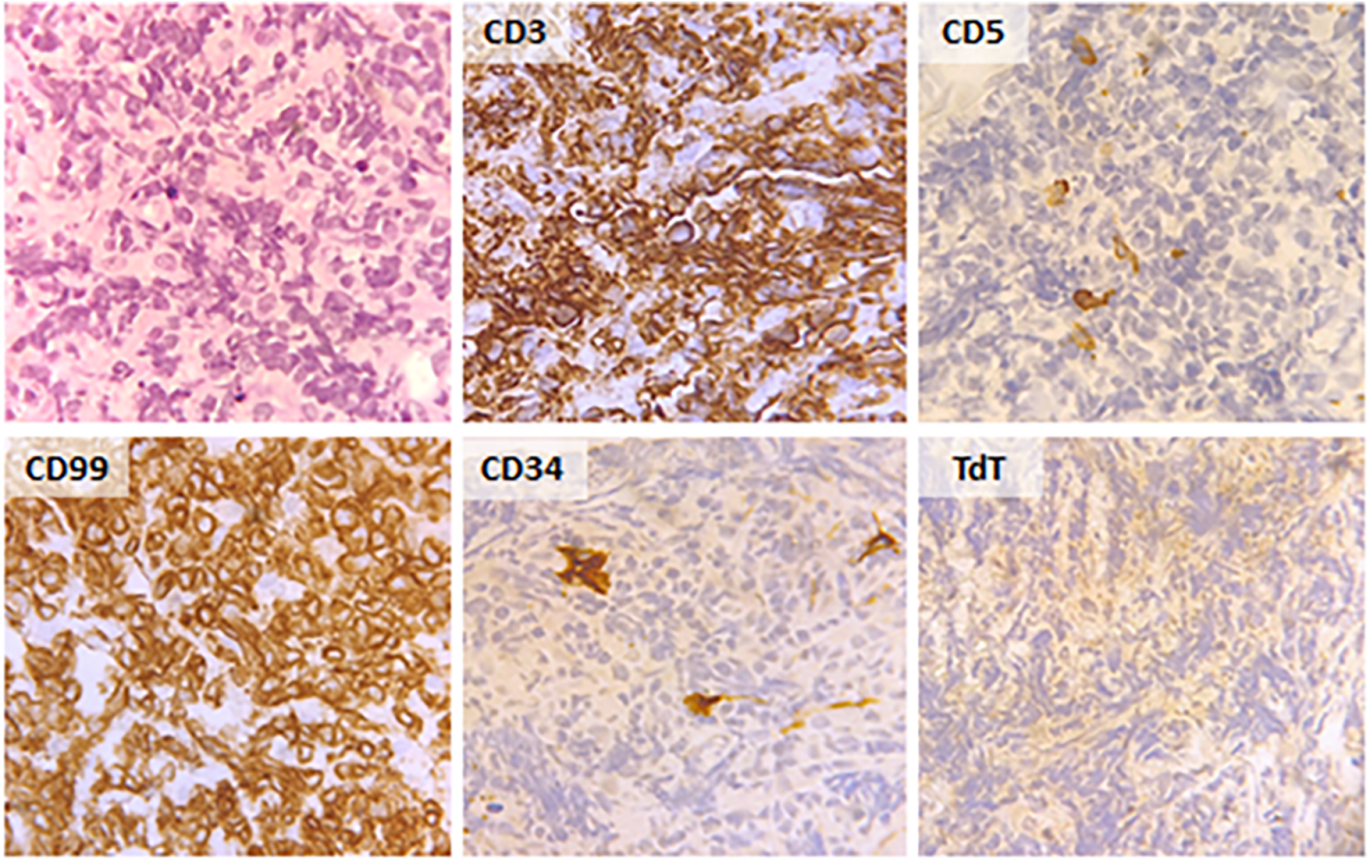
Histological and immunohistochemical findings in the biopsy of the bulky lesion in the superior-anterior mediastinum.

A 57-year-old male was admitted to the General Internal Medicine Department of Padova University Hospital for acute massive haematoma of the left lower extremity associated with macrohaematuria. The patient reported no family or personal history of spontaneous bleeding and haemorrhagic complications, and he was taking no medications at the time of admission. A physical examination confirmed a large haematoma in the left leg and calf extending to the entire limb. A complete blood count revealed only mild normocytic anaemia (haemoglobin 9.3 g/dl; mean corpuscular volume 82.7 fl), with normal leukocytes and platelet count (6.65 × 10^9^/L and 232 × 10^9^/L, respectively). Coagulation parameters at time of admission, performed with fresh citrate plasma, revealed a prolongation of prothrombin time (PT) (21.4 s, n.v. 9.5–13.8), activated partial thromboplastin time (aPTT) (52 s, n.v. 22–32), thrombin time (TT) (34.2 s, n.v. <21) and reptilase time (RT) (60 s, n.v. <21). PT, aPTT and TT were corrected with mixing tests after incubation for two hours (PT mixing test 10.2 s, aPTT mixing test 26 s, and TT mixing test 16 s). The Clauss fibrinogen assay revealed that fibrinogen level, antigen level and activity were 46 mg/dl (n.v. 150–450), 0.70 g/L (n.v. 1.5–4.5) and 26% (n.v. 80–120), respectively. Antithrombin (87%) and von Willebrand factor (vWF:Ag 105 UI/dL) levels, as well as intrinsic (FXII 94%, FXI 113%, FIX 107%, FVIII 92%), extrinsic (FVII 101%) and common (FX 108%, FII 97%) factors of blood coagulation were all within the normal range. Moreover, plasminogen (106%) and α2-antiplasmin (99%) were normal. We used multiple electrode aggregometry (Multiplate® analyzer, Roche Diagnostics, Rotkreuz, Switzerland) to assess platelet function in whole blood, and rotational thromboelastometry (ROTEM® apparatus, Werfen, Bedford, MA, USA) to assess clot formation, strength and lysis in whole blood. In our patient, platelet function was within the normal range (Figure 1, Panel A) and the thromboelastometry profile was compatible with severe hypofibrinogenemia without hyperfibrinolysis (Figure 1, Panel B). In particular, FIBTEM assay which evaluates the contribution of fibrinogen to blood clotting after inhibition of platelet aggregation by cytochalasin D, showed markedly prolonged clotting time (CT 321 s; normal range 38–62 s) which corresponds to the initiation phase of the clotting process; and significantly reduced maximum clot firmness (A10 3 mm; MCF 5 mm, normal range 9–25 mm) which is the maximum amplitude reached in the thromboelastogram. Thrombin generation (ST Genesia, Diagnostica Stago, Asnières-sur-Seine, France) measured in platelet poor plasma showed increased endogenous thrombin potential (ETP 1.31 nM*min, reference range 1.03–1.25). Due to the persistently elevated (mean ± standard deviation) D-dimer levels (2,455 ± 3,467 µg/L, peak 11.701 µg/L, n.v. <250) during hospitalization, the patient underwent venous Doppler ultrasound on the upper and lower extremities which excluded the presence of deep venous thrombosis in both the proximal and distal circulations. Furthermore, a chest computer tomography (CT) scan with and without contrast medium excluded the presence of signs indicative of pulmonary embolism. The results of serum protein electrophoresis were normal, thus excluding the presence of monoclonal gammopathy; Bence-Jones protein was negative. Finally, liver function tests were normal: alanine aminotransferase (ALT) 44 U, n.v. 10–50; aspartate aminotransferase (AST) 25 U, n.v. 10–45; coagulation factor V 105%, n.v. 80–120; albumin 41 g/L, n.v. 35–55; and no underlying infection was detected. The patient was initiated on tranexamic acid 1,000 mg intravenous (Lusofarmaco, Milan, Italy) three times daily and human fibrinogen concentrate (RiaSTAP®, CSL Behring GmbH, Marburg, Germany) 50 mg/kg body weight once daily (tot. 3 gr per day). We observed no improvement of the bleeding symptoms within 5 days of the initiation of fibrinogen replacement therapy at a dosage of 3 gr per day. In particular, two new haematomas appeared on the skin of the back (12 cm and 8 cm in diameter, respectively) and mild haematuria persisted. After administering RiaSTAP®, we observed a rapid increase in plasma fibrinogen levels, albeit followed immediately by a rapid decrease (Figure 2). Given that the chest x-rays findings at the time of admission showed a widening of the mediastinum, especially towards the left with blurring and poor recognition of the aortic profile, the patient underwent a positron emission tomography–magnetic resonance imaging (PET-MRI) scan that showed pathological F-fluorodeoxyglucose (FDG) uptake (maximum standardised uptake value, SUV Max 19) in adrenal glands (left 74 × 90 mm, right 65 × 35 mm), a bulky lesion in the superior-anterior mediastinum (10 cm × 5 cm with a SUV Max 19) and in one right inguinal lymph node (SUV Max 5.2) (Figure 3). Immediately after fibrinogen supplementation, a biopsy of the anterior mediastinum showed a population of immature T-lineage cells, consistent with early T-precursor (ETP) lymphoblastic leukaemia/lymphoma (blasts expressed CD3, CD4, CD7, CD117 and lacked CD2, CD1a, CD5, CD8, CD10, CD20, CD30, CD33, CD34, TdT, with a MIB1 70%–80%) (Figure 4). Bone marrow biopsy was negative for immature cells. This diagnosis prompted admission to the Haematology Unit wherein the patient underwent a lineage-targeted risk-oriented chemotherapy regimen with 5 anti-ALL drugs: induction with vincristine, idarubicin, dexamethasone, cyclophosphamide and pegylated asparaginase according to standard protocols. Pegylated asparaginase was not administered initially due to low plasma fibrinogen levels. The coagulopathy subsided subsequently with normal fibrinogen activity restored a few days after the initiation of induction chemotherapy (Figure 2). No toxicity related to pegylated asparaginase was observed. The patient tolerated post-induction chemotherapy without any further bleeding symptoms or evidence of hypofibrinogenemia. Notably, plasma fibrinogen levels remained consistently within the normal range 6 months after initiating chemotherapy concomitantly with clinical remission of the haematological malignancy.

## Discussion

We report a rare case of early T-cell precursor acute lymphoblastic leukaemia/lymphoma which manifested clinically with severe haemorrhagic diathesis due to severe acquired hypofibrinogenemia — prior to initiating chemotherapy. T-cell lymphoblastic leukaemia (T-ALL) and lymphoblastic lymphoma (T-LBL) are malignancies of immature precursor T cells in the bone marrow and blood (T-ALL), or presenting with primary involvement of thymus, nodal or extranodal sites (T-LBL) (23). Approximately 85%–90% of all lymphoblastic lymphomas are T-LBL. Early T-cell precursor (ETP)-ALL/LBL is a subtype of T-ALL/LBL derived from thymic cells at the ETP differentiation stage that has been reported in 7.4% of adult T-ALL/LBL. It is characterised by a distinct immunophenotypic and genetic profile, with a worse outcome compared with other T-ALL/LBL subtypes. It is quite rare for this type of leukaemia to be associated with haemorrhagic coagulopathy. In our patient, we hypothesised that the two most likely mechanisms underlying acquired hypofibrinogenemia may be fibrinogen sequestration by tumour cells resulting in enhanced removal from the circulation, or consumptive coagulopathy. Several mechanisms support our hypothesis. Firstly, hypofibrinogenemia may have developed as the lead manifestation of the disease and resolved soon after the initiation of induction chemotherapy, thus suggesting the presence of an inverse correlation between tumour cells count and plasma fibrinogen levels in our patient. Notably, fibrinogen replacement therapy prior to the initiation of chemotherapy was not effective as plasma fibrinogen levels dropped again within few hours — the half-life of fibrinogen in healthy subjects is 3, 4 days — thus indicating removal of fibrinogen from the circulation. Secondly, the mixing test was normal and we did not detect the presence of any abnormal proteins on the serum protein electrophoresis, thus excluding an autoimmune mechanism as the cause of hypofibrinogenemia. Thirdly, we performed a full panel of tests that allowed us to exclude other possible causes of acquired hypofibrinogenemia such as the presence of liver diseases; moreover, we also excluded drug-induced hypofibrinogenemia at the time of admission. Interestingly, a case report by Inano S. et al. (20) detected acquired hypofibrinogenemia in a patient with multiple myeloma with a mechanism similar to that postulated in our patient which the authors have coined “leukocyte-mediated fibrinogen removal.” A few other studies have also proposed a mechanism of sequestration by neoplastic cells to explain bleeding diathesis due to acquired von Willebrand syndrome both in patients with solid tumours (24, 25) and in children with Wilms tumour (26–28). It bears noting that the results of functional tests for fibrinogen assessment may have been influenced by elevated D-dimer levels. However, given that both the values observed in the antigen test and in the FIBTEM test fully corroborate the data from the functional tests, we believe that any interference, if ever present, may have been minimal and such as not to significantly alter the results of the tests.

As regards the International Society on Thrombosis and Haemostasis diagnostic criteria for disseminated intravascular coagulation (DIC) (29), the patient had a score of 6 which is indeed suggestive of consumptive coagulopathy. However, we measured all factors involved in both the extrinsic and the intrinsic pathways; furthermore, we also assessed the plasma levels of plasminogen and α2-antiplasmin. All the aforementioned tests came back normal. Moreover, bearing in mind the extent of the haematomas, we cannot definitively exclude a consumptive component which we believe may account for the elevated D-dimer levels. Regarding the thromboelastographic profile, it is worth highlighting that CFT values in EXTEM and INTEM were not more prolonged and that MCF values in EXTEM and INTEM were normal. Given that both CFT and MCF values are a reflection not only of fibrinogen levels but also platelet function and activity, we hypothesised that the increased thrombin generation potential and thus increased platelet reactivity may explain the normal CFT and MCF values observed in our patient. In that regard, it is necessary to mention that A10 values in EXTEM, INTEM and FIBTEM were all below the lower limit of the reference range. These results confirm a state of hypocoagulability due to reduced clot stability, and thus likely to be attributed to the patient's severe hypofibrinogenemia in the first hypothesis.

It is our belief the present study bears two important clinical implications. Firstly, the occurrence of acquired hypofibrinogenemia in the absence of other common causes of acquired hypofibrinogenemia — i.e., liver dysfunction, fibrinogen-specific antibody or drug toxicity — must always raise a suspicion of cancer and prompt a thorough screening. Secondly, a potential adsorption mechanism and/or consumptive coagulopathy that contribute to the removal of fibrinogen from the circulation render fibrinogen supplementation virtually useless as the resolution of the acquired hypofibrinogenemia is strictly dependent on treating the underlying neoplasia.

In conclusion, severe haemorrhagic diathesis due to acquired fibrinogen deficiency is a unique presentation of undiagnosed T-cell acute lymphoblastic leukaemia/lymphoblastic lymphoma, as highlighted in our case. The failure to respond to fibrinogen replacement therapy in the absence of the most common causes of acquired hypofibrinogenemia pointed to two likely mechanisms: (a) adsorption of circulating fibrinogen by tumour cells and (b) consumptive coagulopathy.

## Data Availability

The original contributions presented in the study are included in the article/Supplementary Material, further inquiries can be directed to the corresponding author.
